# Supplementary material for: Facial Synthesis of Adsorbent from Hemicelluloses for Cr(VI) Adsorption
Source: Molecules. 2021 Mar 7;26(5):1443. doi: 10.3390/molecules26051443 (PMC7961731; doi:10.3390/molecules26051443)
Supplement: Supplementary file 1 [file molecules-26-01443-s001.pdf]

Supplementary

Facial Synthesis of Adsorbent from Hemicelluloses for Cr(VI) Adsorption

Yi Wei [1], Wei Chen [1], Chuanfu Liu [1,*], and Huihui Wang [1,*]

[1] State Key Laboratory of Pulp and Paper Engineering, South China University of Technology, Guangzhou China, wygift@163.com (Y.W); geogeo_chen@163.com (W.C.).

* Correspondence: chfliu@scut.edu.cn (C.L.); whh@scut.edu.cn (H.W.); Tel.: +86-20-8711-1735 (C.L.)

Table S1. Specific surface area, pore volume, and pore size distribution of samples

**Sample**	**BET surface area (m^2/g)**	**Average pore size (nm)**	**Total pore volume (cm^3 /g)**
Hemicelluloses	0.97	3.98	0.00
HTC	3.06	2.83	0.002
HTC-NH_2	134.51	20.55	0.69

Table S2. Elements contents of HTC and HTC-NH_2 by XPS analysis

**Sample**	**Element content (%)**		
	**C**	**O**	**N**
**HTC**	**77.83**	21.12	1.04
**HTC-NH_2**	**59.14**	38.77	2.09

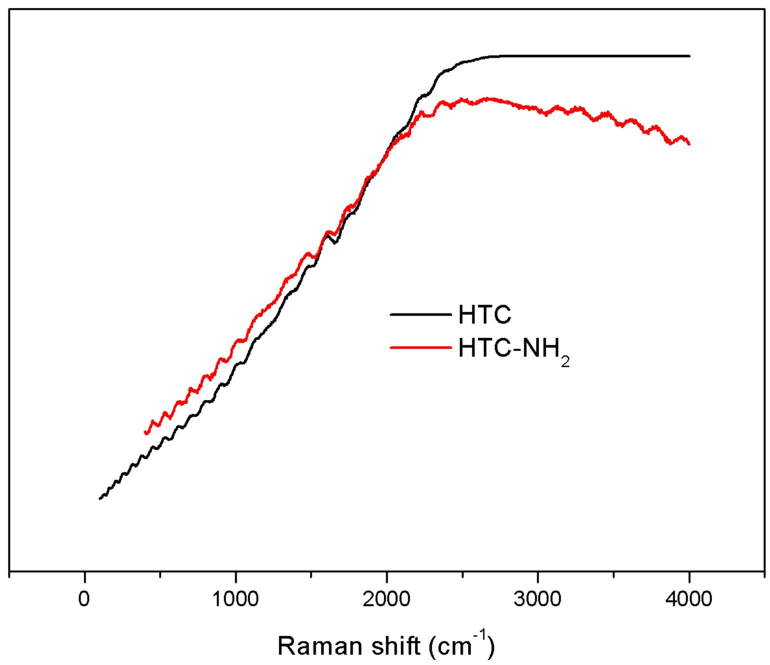

Figure S1. Raman spectra of the hydrothermal carbon materials.

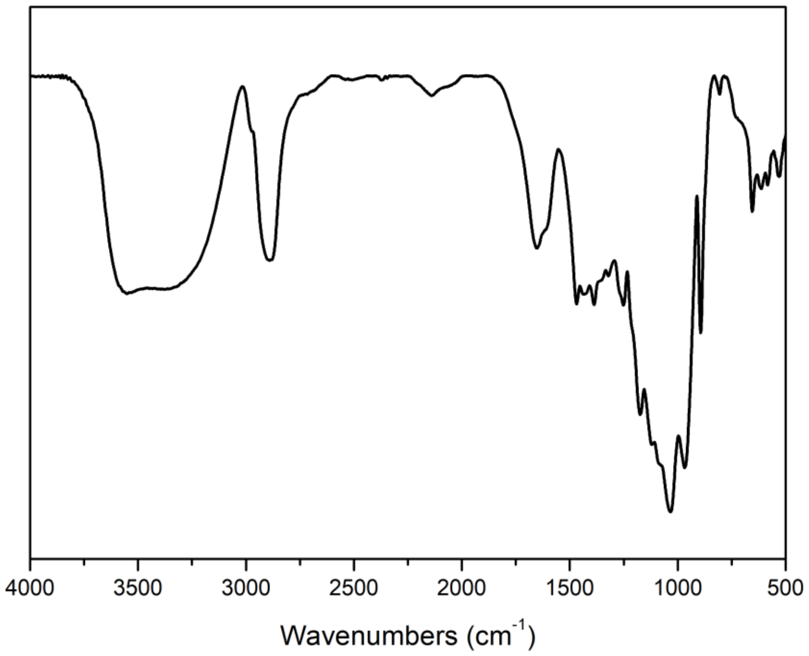

Figure S2. FT-IR spectra of the raw hemicelluloses
